# Computer Vision-Based Bridge Damage Detection Using Deep Convolutional Networks with Expectation Maximum Attention Module

**DOI:** 10.3390/s21030824

**Published:** 2021-01-26

**Authors:** Wenting Qiao, Biao Ma, Qiangwei Liu, Xiaoguang Wu, Gang Li

**Affiliations:** 1 School of Highway, Chang’an University, Xi’an 710064, China; 2017021034@chd.edu.cn (W.Q.); wuxg@chd.edu.cn (X.W.); 2 Inner Mongolia Transport Construction Engineering Quality Supervision Bureau, Hohhot 010020, China; 3 School of Electronic and Control Engineering, Chang’an University, Xi’an 710064, China; 2018232020@chd.edu.cn (B.M.); 2018132015@chd.edu.cn (Q.L.)

**Keywords:** deep convolutional networks, bridge damage detection, expected maximum attention, densely connected networks

## Abstract

Cracks and exposed steel bars are the main factors that affect the service life of bridges. It is necessary to detect the surface damage during regular bridge inspections. Due to the complex structure of bridges, automatically detecting bridge damage is a challenging task. In the field of crack classification and segmentation, convolutional neural networks have offer advantages, but ordinary networks cannot completely solve the environmental impact problems in reality. To further overcome these problems, in this paper a new algorithm to detect surface damage called EMA-DenseNet is proposed. The main contribution of this article is to redesign the structure of the densely connected convolutional networks (DenseNet) and add the expected maximum attention (EMA) module after the last pooling layer. The EMA module is obviously helpful to the bridge damage feature extraction. Besides, we use a new loss function which considers the connectivity of pixels, it has been proved to be effective in reducing the break point of fracture prediction and improving the accuracy. To train and test the model, we captured many images from multiple bridges located in Zhejiang (China), and then built a dataset of bridge damage images. First, experiments were carried out on an open concrete crack dataset. The mean pixel accuracy (MPA), mean intersection over union (MIoU), precision and frames per second (FPS) of the EMA-DenseNet are 87.42%, 92.59%, 81.97% and 25.4, respectively. Then we also conducted experiments on a more challenging bridge damage dataset, the MIoU, where MPA, precision and FPS were 79.87%, 86.35%, 74.70% and 14.6, respectively. Compared with the current state-of-the-art algorithms, the proposed algorithm is more accurate and robust in bridge damage detection.

## 1. Introduction

Bridges play an irreplaceable role in transportation, so their reliability must be guaranteed. Compared with the huge amount of bridge construction costs, bridge repair and maintenance should be periodically estimated. China has the world’s best bridge-building technology, accounting for six of the world’s top 10 sea-crossing bridges, and more than 800,000 highway bridges and 200,000 railway bridges are in use according to the statistics. The safety threat of aging bridges has been regarded as a national public concern, and one of the biggest challenges today is the design of algorithms that can automatically detect bridge damage [1]. In order to prevent large accidents caused by bridge collapses, the bridge structural health monitoring (SHM) [2,3,4] technology has been proposed to evaluate the health of bridges. SHM is built on the bridge big data and the use of various sensors that can monitor the bridge temperature, humidity, wind, deformation, tension and so on [5].

In order to solve the problem of local damage detection of the bridge, the nondestructive detection technology is adopted for key bridge components in practical engineering [6,7]. Several SHMs are capable of large-scale structural detection, but they require a large number of instruments and sensors. To solve this problem, damage detection methods based on computer vision have been developed. Damage detection algorithms based on image processing such as percolation-based image processing [8,9], threshold methods [10,11] and edge detection methods [12,13] are very popular detection methods in the early stages. Chen et al. proposed a method to identify the spatial and temporal distribution of vehicle loads on long-span bridges by using computer vision technology combined with the monitoring information from a dynamic weighing system [14]. Ho et al. introduced an efficient image-based damage detection system that can automatically identify damage to bridge cable surfaces through image processing techniques and pattern recognition [15]. The development of automatic damage detection algorithms is helpful to reduce the maintenance costs and improve the maintenance efficiency of bridges, which has important research value.

The main factors affecting the service life of the bridge are cracks at the bottom of the bridge deck and corrosion of steel components, especially for those that have been used for more than 50 years. The existence of cracks will shorten the service life of bridges and increase the probability of accidents. Therefore, it is of great value to study bridge damage detection. Because of the large amount of traffic on the bridge, it is very dangerous to detect the damage directly in the field [16,17], and it is time-consuming and laborious for a person to search for cracks from thousands of pictures. As a result, many researchers have tried to develop automatic bridge damage detection technology [18,19]. Image-based crack detection algorithms have made great progress in recent years, but the reason why they can’t be applied effectively in practice is that the detection accuracy of these algorithms is very low in the face of complex and changeable environment.

**Figure 1:**
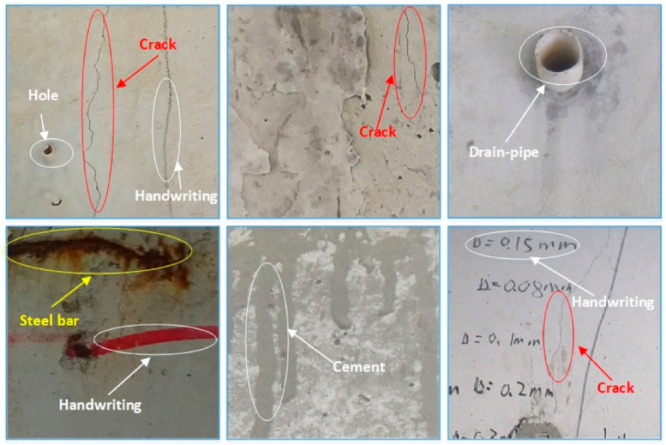
Some images taken under a bridge (including cracks, exposed steel bars and other features).

Different from cracks in ordinary pavement, the cracks at the bottom of a bridge are smaller and the influence of other features is larger, as shown in Figure 1. These features mainly consist of holes, exposed and rusted steel bars, drain-pipes and traces of uneven cement. Some of these noises are similar in shape to cracks and are easily detected as cracks by mistake. In addition, pen marks are used to indicate the cracks when the images are collected, which also have a high degree of similarity with cracks. Due to the complexity of the various features, most crack detection algorithms are not accurate and effective enough. As a result, the automatic detection of the length and width of cracks on bridge pavements is a challenging task. Deep learning algorithms are used to detect bridge cracks mainly because of their robustness and learnability. Robustness means that the deep learning algorithm has a stable ability to extract features of cracks from the images, and then identify and locate cracks according to these features. This study aims to accurately detect cracks and exposed steel bars in complex environments using deep neural networks. Deep learning has two apparent advantages in damage detection: robustness and automation [20]. Robustness refers to the ability to stably extract features from images, and then to identify and locate damaged areas based on these features. Automation means that the feature extraction process is automatic or semi-automatic. With these two features, deep learning can distinguish different types of damage from the background in images. Therefore, it is appropriate to identify bridge cracks and steel bars using deep learning methods.

In recent years, the development of deep learning in the field of object detection has broken through the concept of traditional image processing technology and achieved excellent results [21,22,23]. The same is true for damage detection, where deep neural networks have brought unprecedented improvements, which makes it possible to realize crack detection in complex environments. Dorafshan et al. compared the performance of common edge detectors and deep convolutional neural networks (DCNNs) for image-based crack detection in concrete structures [24], and the result was that a CNN-based crack detector has much better performance than a traditional edge detector. Research on crack detection and recognition has grown rapidly because of the development of CNN. As long as a large number of multi-type data are used to train the neural network, the features of cracks can be learned and the cracks can be detected more accurately. Wang et al. proposed a CNN-based crack detection network called CrackNet [25] and its improved version CrackNet II [26], both of which can detect cracks in 3D road images with good results and fewer network parameters. Cha et al. trained a CNN to divide images of concrete buildings into cracked and non-cracked areas using a sliding window scheme [27]. CNN has great research significance because it has great variability, and networks with different depths are suitable for different situations. Hui et al. added bypass channels into the ordinary CNN, fused the features of all channels, and achieved better accuracy in crack detection of bridge steel box girders than with the original CNN [28]. The proposals of ResNet [29] and DenseNet [30] have injected new vitality into the field of deep learning, as both of them change the structure of the traditional CNN that transmits information only from one layer to the next layer. DenseNet requires less than half the parameters compared to ResNet, but achieves the same accuracy. For industry applications, DenseNet can significantly save bandwidth and reduce storage overhead. In fact, DenseNet is more efficient than other networks. The key to DenseNet is the reduction of the required computation per layer of the network and the reuse of features. Each layer of DenseNet only needs to learn a few features, resulting in a significant reduction in the number of parameters and computations. DenseNet has a very good anti-overfitting performance. Compared with the classifier of the general neural network which directly depends on the features of the last layer of the network, DenseNet can make comprehensive use of the features of the low complexity of the shallow layer, so it is easier to obtain a smooth decision function with better generalization performance. In the field of semantic segmentation, the fully convolutional networks (FCNs) [31] and U-net [32] have made waves again, because they use deconvolution instead of a full connection layer to achieve end-to-end pixel level segmentation. The encoder-decoder network (SegNet) [33] concept changed the way of up-sampling on the basis of FCN, and it has better performance in processing time and memory occupancy. These methods were soon applied to crack detection and have achieved remarkable results [34,35,36].

Deep learning does not function alone in the field of crack detection, it can also be combined with other image processing technologies to achieve better results [37]. Chen et al. combined a convolutional neural network with naive Bayes to detect cracks in nuclear inspection videos [38], where the false positives are subtly removed by the use of the naive Bayes method. Song et al. introduced a new multiscale extended convolution module, which can learn plentiful deep features and make the acquired features more recognizable in complex backgrounds [39]. It is worth highlighting that with the development of computer vision techniques, especially deep learning, automatic object detection technology has been greatly improved. What we need to do is to further study on the basis of deep learning and design a scheme that can be applied to actual bridge damage detection work. Attention is widely used for various tasks, such as machine translation and video classification [40,41]. EMANet [42] re-thought the attention mechanism from the perspective of EM algorithm and calculated the attention map by iteration. It transformed the attention mechanism into an expectation-maximization manner and iteratively estimated a more compact base upon which to compute attention maps; this module is robust and takes up less memory. The approach in this paper is motivated by the above works, and we introduce the EMA module into DenseNet to make the detection algorithm pay more attention to the damaged parts of the bridge. The main contributions of this paper may be listed as follows:

(1) A novel bridge damage detection method based on densely connected convolutional networks with expectation maximum attention module (EMA-DenseNet) is proposed, which can detect cracks and exposed steel bars efficiently in the complex environment at the bottom of the bridge.
(2) The structure of the advanced DenseNet was redesigned and the EMA module was added in the last pooling layer of the DenseNet, which is obviously helpful for the damage feature extraction.
(3) Experiments were carried out on a public crack dataset and a bridge damage dataset respectively and the results were compared with the most advanced algorithms, showing the superiority of the proposed method.

## 2. Data Collection

As mentioned in Section 1, the damage at the bottom of the bridge mainly includes cracks and exposed steel bars. At present, there are some crack datasets but no public bridge damage dataset. Therefore, we use both a crack data set and a bridge damage dataset made by us into account.

### 2.1. Public Crack Dataset

**Figure 2:**
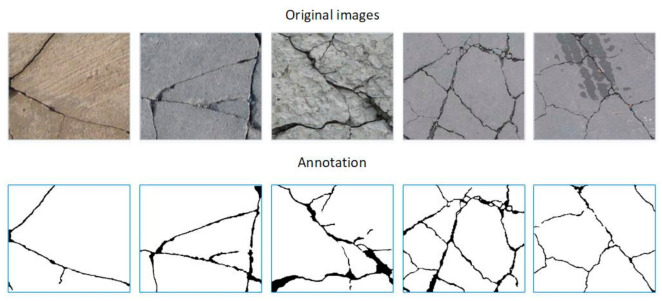
The public concrete crack dataset collected by Yang et al.

Yang et al. [34] collected more than 800 crack images of concrete pavement, and here we use this crack dataset to verify the effectiveness of the proposed method. The original images were formatted as JPG, while the resolution ranged from 72 to 300 dpi. Figure 2 shows some samples of this dataset, where the morphology of the cracks is varied, but the noise in this data set is relatively low. In the ground truth images, the white area with the pixel value of 0 represents the background, and the black area with the pixel value of 255 represents a crack.

### 2.2. The Established Bridge Damage Dataset

**Figure 3:**
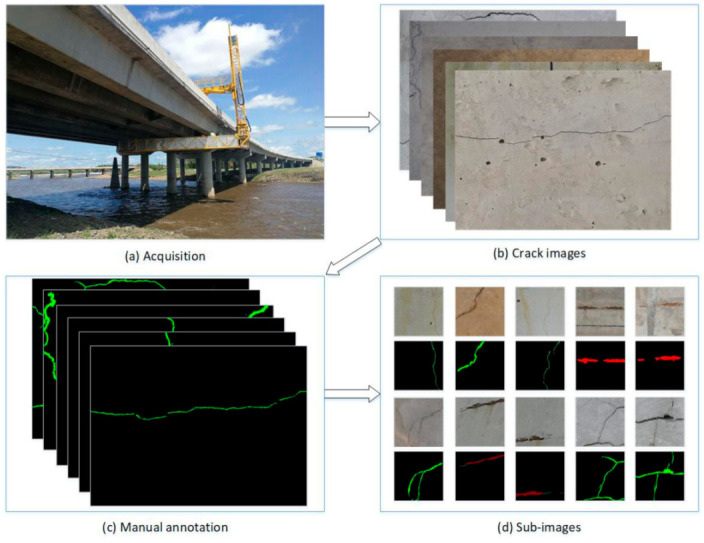
The establishment of bridge damage dataset consists of the following parts: (**a**) bridge image acquisition, (**b**) the captured bridge images, (**c**) manually marking the damage pixel area and (**d**) image clipping.

The bridge crack dataset described above is relatively simple, and there are few interference factors in the images, which may be inconsistent with the actual detection environment. Therefore, we collected many bridge images including cracks and exposed reinforcement from several different bridges in Xuzhou (Zhejiang Province, China). As shown in Figure 3a, the bridge images can be obtained using a standard bridge inspection vehicle. The image shooting position can be kept at a constant distance from the bridge, which will greatly facilitate the detection of damage. A total of 400 images, with resolution of 4464×2976 pixels, were captured using a 5D Mark IV digital single lens reflex camera (Canon, Zhuhai, China). Since the deep network needs labeled images for supervised learning, we carefully marked the collected images at the pixel level. Figure 3b,c show part of the original images and the manually annotated images, where red represents rebar pixels and green represents cracks. The proposed network can handle any image size however, training large images may lead to excessive use of GPU memory, leading to training failure. To avoid this situation, the original images were cropped to a size of 480×480. There’s another advantage to doing this, in that the proportion of cracks in the image is increased, which is more conducive to improving the detection accuracy. Since most of the sub-images after cropping are images without any damage, we only selected images with bridge damage, including 1800 crack images and 2500 rebar images. Data enhancement is necessary to enhance the training effect, so all images were rotated 90, 180, and 270 degrees, respectively. It is worth noting that in the data collection stage, we only annotated, clipped and augmented the collected images. During the data annotation process, the areas containing bridge damage was marked as cracks or exposed bars, and the other area (containing various noises) was marked as the background. In this way, the deep learning network model can learn the different characteristics of the bridge damage and the background during training, so as to eliminate the noise.

## 3. Methodology

**Figure 4:**
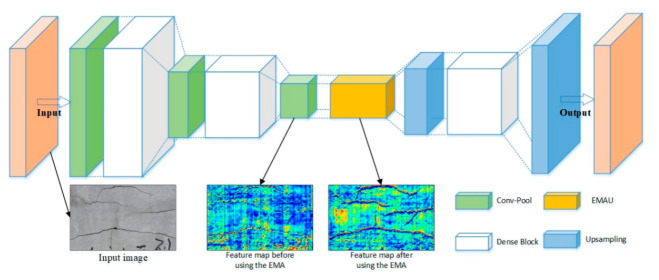
Overall architecture of the proposed EMA-DenseNet.

The convolutional neural network has a very good performance in extracting features, while the use of deconvolution enables the features to map to the input size, so as to achieve a pixel-level prediction. In this section, the network structure of the EMA-DenseNet will be introduced, and the architecture of the network is shown in Figure 4. The advanced DenseNet is used as the backbone to extract the features of the input images, then the expectation-maximization attention (EMA) module is adopted to obtain more detailed features. As can be seen from Figure 4, after the adoption of EMA module, the fracture characteristics are clearer in the feature map. We enlarge the feature map to the same size as the input by up-sampling layer, and finally we adopt Sofmax layer for pixel-level classification. Below, we will detail the structure of the proposed framework.

### 3.1. The DenseNet Backbone

**Figure 5:**
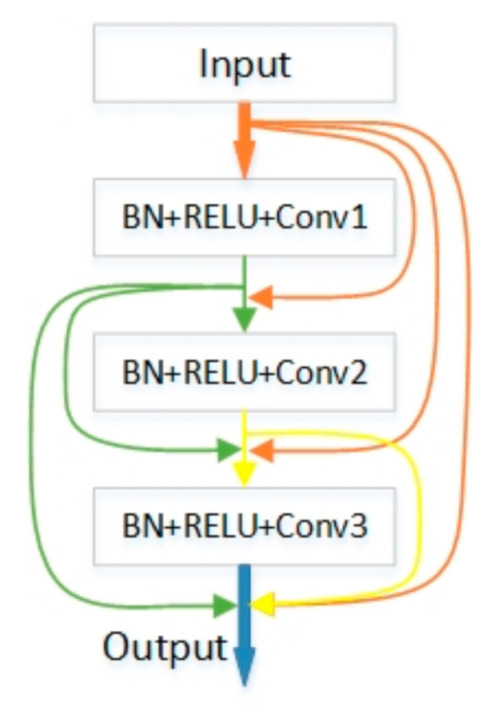
The basic schematic of a 4-layers dense block.

Unlike a traditional convolutional neural network which simply passes information on to the next layer, DenseNet [30] has designed a more densely connected block, which is called dense block. Figure 5 shows the configuration principle of the dense block, each convolutional layer in each block accepts the output of all previous layers in the Bblock. Here, we use $x_{0}$ to represent the input layer, $x_{l}$ for the output of the other layers, where L is the number of layers. Then the output of each layer can be defined as:

(1) $$x_{l}=\;H_{l}\left(\left[x_{0},x_{1},\ldots ,x_{l-1}\right]\right)$$

where $\left[x_{0},x_{1},\ldots ,x_{l-1}\right]$ represents the channel-wise concatenation of the feature-maps produced in layers 0, . . . , l−1, and $H_{l}$ refers to the operation of the sequence BN-ReLU-Conv. The DenseNet enhances gradient back propagation due to dense connections, making the network easier to train.

Figure 5 just shows the basic structure of a dense block and depending on the number of convolutional layers in the dense blocks, there are four DenseNets with different network depths. In this paper, the EMA-DenseNet employs three dense blocks, one less than DenseNet-121. Two dense blocks are placed on the downsampling path and one on the upsampling path. We did not use the Softmax layer at the last layer of DenseNet for classification because the cracks and steel bars need to be segmented and extracted. The conventional solution is to directly use deconvolution layer to obtain the feature map of the same size as the input for prediction. However, this paper realizes the deficiency of CNN and uses an expectation-maximization attention (EMA) module to further improve the quality of feature maps.

### 3.2. Expectation-Maximization Attention Module

**Figure 6:**
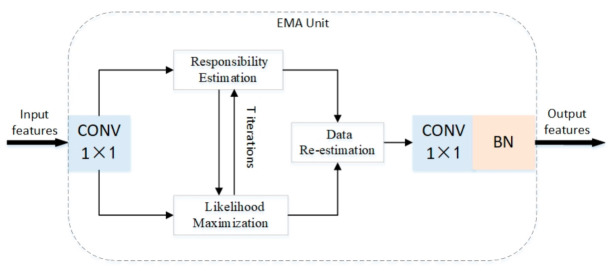
Structure of the expectation-maximization attention unit (EMAU).

The normal Non-Local module selects all data points as the bases, but the EMA module aims to obtain a compact base set through an EM algorithm [42]. The overall structure of the EMA module is shown in Figure 6. For simplicity, we assume that the single image after passing through DenseNet produces a C×H×W feature map X. Firstly, a convolution operation is carried out without ReLU activation function, and the input value range is transformed from $\left(0,+\infty \right)$ to $\left(-\infty ,+\infty \right)$. The last 1×1 convolution is inserted to convert the re-estimated $\tilde{X}$ into the residual space of X.

The operation of the EMA module consists of the following three steps:

*Step 1*: Responsibility estimation. We reshape X into N×C, where N=H×W, given the input $X\in {\mathbb{R}}^{N\times C}$ and the initial bases $\mu \in {\mathbb{R}}^{K\times C}$. This step computes the expected value of $z_{nk}$, which corresponds to the responsibility of the k-th basis μ to $x_{n}$, where 1<k<K and 1<n<N. Then use λ as a hyper-parameter to control the distribution of Z, the operation of the k-th iteration can be formulated as:

(2) $$Z^{\left(t\right)}=softmax\left(\lambda X{\left(\mu^{t-1}\right)}^{⊺}\right)$$

*Step 2*: Likelihood maximization. This step updates μ by maximizing the complete data likelihood with the estimated Z. In order to keep the bases in the same embedding space as the input X, the bases µ is updated using the weighted sum of X. So in the t-th iteration, $\mu_{k}$ is calculated by:

(3) $$\mu_{k}^{\left(t\right)}=\frac{z_{nk}^{\left(t\right)}x_{n}}{\sum_{m=1}^{N}z_{mk}^{\left(t\right)}}$$

The EM algorithm executes Step 1 and Step 2 alternately until the convergence criterion is satisfied. 

*Step 3*: Data re-estimation. When the iteration of EM algorithm is finished, the final $\mu^{\left(t\right)}$ and $Z^{\left(t\right)}$ are used to re-estimate the X, namely $\tilde{X}$. $\tilde{X}$ is very compact in the feature space, and the characteristic variance inside the object is smaller than the input characteristic variance. $\tilde{X}$ is formulated as:

(4) $$\tilde{X}=Z^{\left(T\right)}\mu^{\left(T\right)}$$

### 3.3. Loss Function

The design of loss function will have a great impact on the performance of the network. A good loss function can make the training get twice the results with half the effort. Most of the previous [43,44] methods use the cross-entropy loss function to calculate the accuracy of each pixel prediction, as shown in Equation (5). Here, $c\;=\;\left\{0,\;1,\;2\right\}$ represents the category of each pixel, while y is the true value and $\hat{y}$ is the predicted result. This loss function focuses on determining the category of each pixel, but does not focus on the relationship between pixels:

(5) $$Loss=-\sum \sum_{c=0}^{n}y_{c}log{\hat{y}}_{c}$$

In order to better solve the problem of bridge damage detection, Mei et al. [45] treated the pixel-level crack detection as a connectivity problem. First, the binary mask annotation was transformed into a connectivity map, eight connectivity maps can be generated based on the binary mask information. The loss function is designed to optimize the neural network parameters so that all eight connectivity maps are closer to the real value. This loss function is the sum of the cross-entropy functions of all eight connectivity maps, as shown in Equation (6).

(6) $$Loss=\sum_{k=1}^{8}$$

where $y_{A_{k}}\left(i,j\right)$ is the true label of a pixel at location i and j in the connectivity map $A_{k}$, and ${\hat{y}}_{A_{k}}\left(i,j\right)$ is the predicted result. However, this loss function is only for crack detection, we extend it to multiple damage detection. The new loss function will be formulated by Equation (7):

(7) $$Loss\;=\;-\sum_{i,j\in image}\sum_{i=1}^{n}\sum_{k=1}^{8}y_{A_{k}}\left(i,j\right)log{\hat{y}}_{A_{k}}\left(i,j\right)$$

## 4. Results and Analysis

### 4.1. Model Training

The training process was carried out on the high-performance computing platform of Chang’an university, which has four Tesla V100-SXM2 GPUs. We implement our EMA-DenseNet using Pytorch, which is an open-source platform for deep learning. Due to the large image size, training the EMA-DenseNet requires a large amount of memory, which will lead to a heavy burden for the training process. In addition, the crack area takes up a small proportion in the whole image, and many background areas are meaningless to the training process. Therefore, the original bridge crack images are divided into several small patches with a size of 480×480. Since the image size of the open crack data set is different from that of the bridge damage data set in this paper, the batch size for training on these two data sets is different. Through a lot of experiments, we finally set the batch size as 16 and 12, respectively, in the training process of crack dataset and bridge damage dataset.

To optimize loss functions, we chose the powerful Adam optimizer [46], which has faster convergence than Momentum, RMSprop, etc., and the initial learning rate is set to 0.0001. The momentum and weight decay are set to 0.9997 and 0.0005, respectively. In the beginning, we initialize $\mu^{\left(0\right)}$ using Kaiming’s initialization method [47]. All the normalization operations in the experiments adopt the synchronous batch normalization method. According to [42], we set the default parameter K = 64, λ = 1, the number of iterations T = 3. The $\mu^{\left(T\right)}$ can be generated after iterating over an image, we average it over a mini-batch to get the ${\bar{\mu }}^{\left(T\right)}$. The $\mu^{\left(0\right)}$ will be updated using the moving average as follows:

(8) $$\mu^{\left(0\right)}\leftarrow \alpha +\left(1-\alpha \right){\bar{\mu }}^{\left(T\right)}$$

where the momentum $\alpha \;\in \;\left[0,1\right]$. In order to ensure the stable update of $\mu^{\left(t\right)}$, we apply Euclidean normalization upon it. 

### 4.2. Evaluation Metrics

To demonstrate the feasibility of the proposed scheme, we compared our EMA-DenseNet with FCN [34], SegNet [33], DeepLab v3+ [48] and SDDNet [49]. During the training, the metrics between the predicted results and the ground truth is calculated at the end of each iteration. Assuming there are k + 1 classes, $p_{ij}$ represents the number of pixels that belong to class i but are predicted to be class j, then we can calculate these metrics: pixel accuracy (PA), mean pixel accuracy (MPA), mean intersection over union (MIoU) and precision by Equations (9)–(12).

PA represents the proportion of the predicted number of pixels in the total number of pixels, which is defined as:

(9) $$PA=\;\frac{\sum_{i=0}^{k}p_{ii}}{\sum_{i=0}^{k}\sum_{j=0}^{k}p_{ij}}$$

MPA is a simple upgrade of PA. We calculate the proportion of pixels in each class that are correctly classified, and then take the average of all classes. It is formulated as:

(10) $$MPA=\frac{1}{k+1}\;\sum_{i=0}^{k}\frac{p_{ii}}{\sum_{j=0}^{k}p_{ij}}$$

MIoU is a standard measure of semantic segmentation techniques, which takes the average value after calculating the cross over ratio on each class. It is defined as:

(11) $$MIoU=\frac{1}{k+1}\;\sum_{i=0}^{k}\frac{p_{ii}}{\sum_{j=0}^{k}p_{ij}+\sum_{j=0}^{k}p_{ji}-p_{ii}}$$

Precision is defined as the percentage of correctly identified damage pixels with relative to all detected pixels, which is calculated as:

(12) $$Precision=\;\frac{1}{k+1}\;\sum_{i=0}^{k}\frac{p_{ii}}{\sum_{j=0}^{k}p_{ji}}$$

### 4.3. Results on the Public Crack Dataset

**Figure 7:**
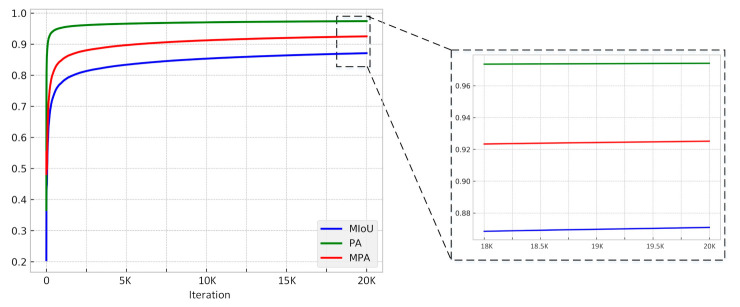
The MIoU, PA and MPA of the proposed EMA-DenseNet on the public crack dataset during each iteration, the figure on the right is a magnification of the interval on the left.

**Figure 8:**
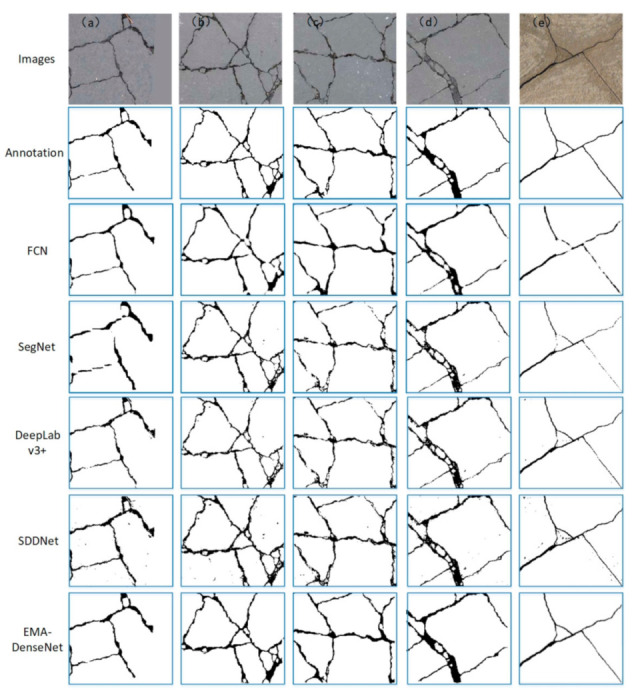
Predicted results compared with ground truth, the images (**a**–**e**) are randomly selected from the test set.

The PA, MIoU, and MPA will be calculated during the training, and Figure 7 shows the process of the training. In the first 2000 iterations, these three indexes increased exponentially, this indicates that our model converges very quickly. The right figure of Figure 8 is a larger version of the last 2000 iterations, where the MIoU grew slowly and finally reached a stable value of 87.42%. The training process proves that the proposed algorithm is reliable. We compared the final stability model with the FCN, SegNet, DeepLab v3+ and SDDNet, Table 1 presents the MIoU, PA, MPA, and precision for these methods. For the proposed method they are 87.42%, 97.58%, 92.59% and 81.97%, respectively. PA is very high because the percentage of damaged areas in the picture is particularly low. MPA is the mean pixel accuracy of various categories, and it is usually more convincing than PA. Obviously, four of the five evaluation indicators of our proposed algorithm are the highest, with only PA slightly lower than FCN. This result shows that the EMA-DenseNet has better performance than other methods in concrete crack detection. Finally, the processing speed of each method is presented. The processing speed is related to the number of parameters in the network, DeepLab v3+ has the slowest processing speed and can predict 12.8 images in one second. Due to fewer parameters, the SDDNet has the fastest prediction speed, with a FPS of 33.2. The proposed EMA-DenseNet also has some advantages in processing speed, only a little lower than SDDNet, ranking the second.

Several images were randomly selected from the test set for detailed comparison, Figure 8 presents the visual comparisons of the crack detection results using these five methods. All the images in the first row are the original images to be detected, the second line are the manually annotated images and the following are the prediction results of different methods. A total of five different crack images were selected, and the fracture morphology in each image was relatively complex. It can be seen from Figure 8 that the EMA-DenseNet is a more powerful network, which makes it better for noise processing. The experimental results show that these methods can detect cracks well, but the predicted crack pixels of SegNet and FCN are discontinuous. On the other hand, the prediction results of SDDNet and DeepLab v3+ algorithm contain many noise points, while the proposed EMA-DenseNet is closer to the ground truth.

It is not convincing to subjectively judge whether the predicted results are good or bad. For the prediction results of these four pictures, we calculated the MIoU of each method respectively, as shown in Table 2. By comparison, it can be seen that the MIoU of the proposed EMA-DenseNet prediction results are slightly higher than other methods. This comparison proves the effectiveness of the method presented in this paper, but the reason why there is not much difference is that the contrast ratio of cracks in this dataset is relatively high and easy to distinguish. Therefore, we do the same comparison on the bridge damage dataset in the next section.

### 4.4. Results on the Bridge Damage Dataset

**Figure 9:**
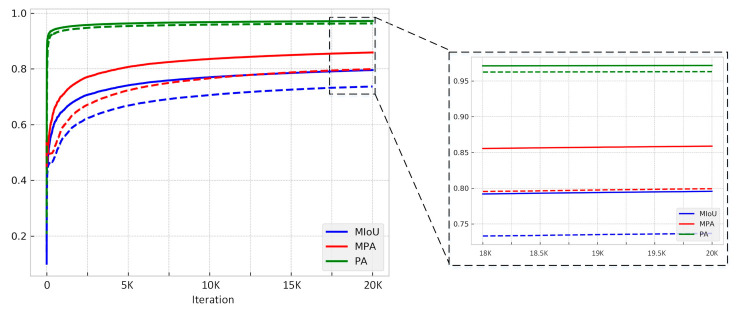
The MIoU, PA and MPA of the proposed EMA-DenseNet on our bridge damage dataset during each iteration, the solid lines indicate the proposed networks with EMA module, and the dotted lines represent networks without EMA modules.

To further verify the proposed method, a more challenging bridge damage dataset collected by our team is employed. The surface of the bridge is damaged due to the deformation of the steel bars, which is generally relatively wide and has some cracks around it. Due to the influence of rain, the exposed steel bars are basically in a state of rusting, which also belongs to a kind of bridge damage. To demonstrate the effectiveness of the EMA module, two types of training were carried out here, one with the EMA module and the other without the EMA module. Figure 9 shows the performance curve when the training finished, the solid and dotted lines represent the adoption and non-adoption of EMA modules, respectively. Similarly, these three indicators also reach a high level very quickly, indicating that the convergence process of the algorithm is very short. At the end of the training, the MIoU, PA and MPA of the proposed EMA-DENSENET reached 79.87%, 97.31% and 86.35%, respectively, while those of the network without EMA module were 73.65%, 96.31%, and 79.91%, respectively. Table 3 lists the MIoU, PA, MPA, and Precision for different methods on the bridge damage dataset. SDDNet and DeepLab v3+ perform better than FCN and SegNet, but are still not comparable to the proposed approach. The biggest difference is MPA, the EMA-DenseNet is 2.63% higher than FCN and 4.20% higher than SegNet. In terms of the speed of recognition, the image size of the bridge damage dataset is larger, so the FPS of all methods decrease greatly. Even so, the processing speed of these five methods is relatively fast. SDDNet has the highest FPS, followed by the proposed algorithm. However, the accuracy of SDDNet is rather lower than ours. This suggests that our algorithm is more suitable and more robust for detecting damage in complex environments.

**Figure 10:**
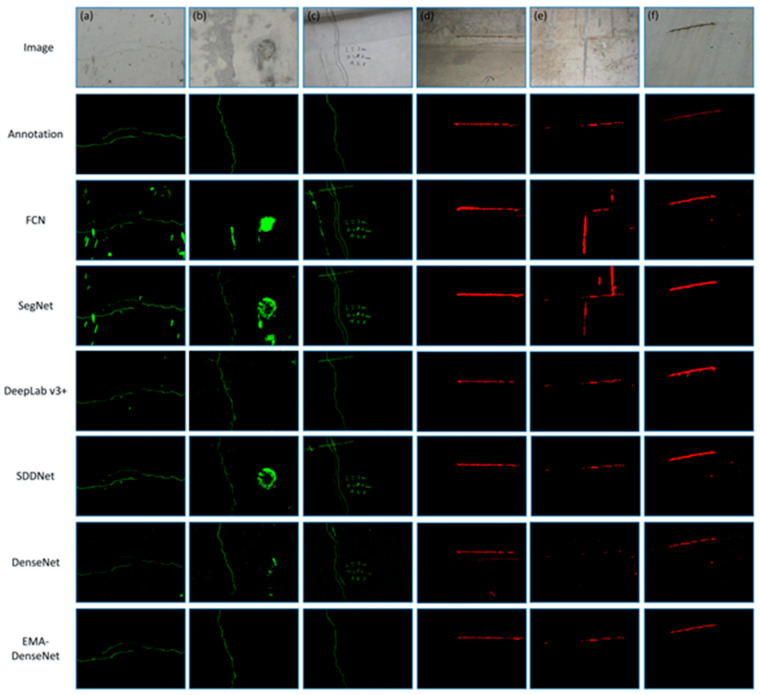
Prediction results for some bridge damage images, the images (**a**–**f**) are randomly selected from the test set.

Some sample images in the test set are used to further analyze and compare, the original images and predictions of different methods are shown in Figure 10. As can be seen from the original image, the bridge images are particularly fraught with interference factors. Most of the noise in these images is spots, which are easily mistaken for cracks by traditional methods. This dataset can reflect the advantages and disadvantages of each method. Figure 10a–c are the crack images, and the Figure 10d–f are the steel bar images. It can be clearly seen that many algorithms are not effective in detecting the bridge damage. For example, when the FCN and Segnet detected cracks in Figure 10a, some spots that are not cracks are detected as cracks. There are some lines and words beside the cracks in Figure 10c, which are very similar to the crack. The FCN, SegNet and SDDNet mistakenly recognize some lines or words as cracks, while the DeepLab v3+ and the proposed EMA-DenseNet accurately detected the cracks. Among the six images, the easiest to detect is Figure 10d. Although there are some noise points around the exposed steel bar, the damage area is more obvious and easy to detect, so all the methods have good detection. The rebar in Figure 10e is also not easy to detect, FCN and SegNet detected some cement traces as rebar. The prediction results of DenseNet without EMA module are also given here. The performance of DenseNet were also good, but there were a lot of noise. The damage region predicted by EMA-DenseNet is more complete and has less noise. By comparing the two cases, it can be found that the application of EMA module greatly improves the performance. The prediction results show that the proposed method has better noise processing ability and robustness in the case of simultaneous identification of multiple damages. It should be acknowledged that some damage segments are still not correctly identified in our method, the effective solution is to increase the number of images in the training.

Similarly, we also analyzed the MIoU of these six images separately, as shown in Table 4. FCN failed to detect cracks in image (b) so that it has the lowest average MIoU. For the predicted results, the average MIoU of the proposed algorithm is 80.4%, which is higher than the other four methods. DeepLab V3+ ranked second with an average MIOU of 79.0%. The MIoU of DeepLab V3 + is significantly higher than the other three algorithms, but 1.4% lower than our EMA-DenseNet. Through the comparison, it is shown that the proposed algorithm has better performance in the prediction of these six images, which indicates that this study has certain significance. Based on the above analysis, it can be seen that the bridge damage have complex characteristics, and the method in this paper has great advantages in the detection of cracks and the exposed steel bars due to the use of dense block and expected maximization attention module. Although the proposed method has achieved the most advanced performance, the current method still has some limitations. For example, the algorithm needs to perform cutting operations when detecting images with high resolution, otherwise the effect is very poor.

## 5. Conclusions

This paper presents a novel algorithm called EMA-DenseNet for the detection of multiple damage on the bottom of bridges. The proposed framework can automatically detect cracks and exposed steel bars against the complex background of the bridge. We adjusted the structure of the DenseNet by adding an upsampling layer so that it can achieve pixel-level prediction. In addition, behind the last pooling layer of the network, we adopted an EMA module to iterate the features acquired by the subsampling path, making it more sensitive to cracks and reinforcement pixels. Moreover, the validity of the EMA module is verified by the comparison of feature map, the obtained feature map has strong robustness and has great suppression to noises. More importantly, a new loss function is adopted to train the proposed network, which pays more attention to the connectivity of the damage area. One of the datasets we used is the concrete surface crack dataset, the other is the bridge damage dataset we collected using a camera. Both datasets were manually annotated by professionals, with 80% used for training and 20% for validation.

On the public crack dataset, the performance of the proposed algorithm is slightly higher than FCN, SegNet, DeepLab v3+ and SDDNet. While on the bridge damage dataset, the MIoU and MPA of EMA-Densenet are much higher than with these four algorithms. Specifically, this algorithm can quickly detect cracks and steel corrosion on the bottom of bridges, while the accuracy and processing speed of other algorithms are relatively low. In conclusion, the model has strong stability and robustness, which can solve the interference of the complex environment at the bottom of the bridge to damage detection. The good performance of the proposed network provides a possibility for large area automatic damage detection, it can be applied to bridge damage detection.

## Figures and Tables

**Table 1:** Comparison of Performance for Different Methods on Public Concrete Crack Dataset.

Method	MIoU (%)	PA (%)	MPA (%)	Precision (%)	FPS (f/s)
FCN	85.77	97.96	90.12	81.73	15.6
SegNet	85.35	96.58	88.30	78.55	18.5
DeepLab v3+	86.50	97.29	91.57	81.08	12.8
SDDNet	85.27	96.91	91.01	81.50	33.2
Ours	87.42	97.58	92.59	81.97	25.4

**Table 2:** Mean intersection over union (MIoU).

Image	FCN	SegNet	DeepLab v3+	SDDNet	Ours
(a)	87.0%	83.1%	87.3%	85.8%	87.3%
(b)	84.2%	85.2%	85.2%	84.3%	85.9%
(c)	82.2%	86.5%	85.9%	85.1%	86.8%
(d)	84.7%	83.1%	87.0%	84.5%	87.8%
(e)	83.6%	83.0%	87.3%	86.0%	88.1%

**Table 3:** Comparison of Performance for Different Methods on Bridge Damage Dataset.

Method	MIoU (%)	PA (%)	MPA (%)	Precision (%)	FPS (f/s)
FCN	74.75	94.05	81.72	71.54	8.1
SegNet	75.03	93.28	82.15	71.67	9.0
DeepLab v3+	78.86	95.78	85.33	74.71	7.4
SDDNet	77.10	94.08	83.92	73.78	18.5
Ours	79.87	97.31	86.35	74.70	14.6

**Table 4:** Mean intersection over union (MIoU).

Image	FCN	SegNet	DeepLab v3+	SDDNet	Ours
(a)	75.7%	75.9%	78.8%	77.0%	80.5%
(b)	61.6%	75.2%	79.8%	77.1%	81.9%
(c)	73.2%	74.9%	79.5%	74.4%	80.3%
(d)	78.7%	80.1%	80.5%	80.6%	81.8%
(e)	74.6%	72.9%	77.0%	77.2%	78.3%
(f)	78.7%	79.3%	78.4%	78.1%	79.3%
mean	73.8%	76.4%	79.0%	77.4%	80.4%

## References

[1] La H.M., Gucunski N., Dana K., Kee S.-H. (2017). Development of an autonomous bridge deck inspection robotic system. J. Field Robot..

[2] Jeong S., Hou R., Lynch J.P., Sohn H., Law K.H. (2017). An information modeling framework for bridge monitoring. Adv. Eng. Softw..

[3] Worden K., Cross E. (2018). On switching response surface models, with applications to the structural health monitoring of bridges. Mech. Syst. Signal. Process..

[4] Zhou G., Li A., Li J., Duan M. (2018). Structural Health Monitoring and Time-Dependent Effects Analysis of Self-Anchored Suspension Bridge with Extra-Wide Concrete Girder. Appl. Sci..

[5] Guo A., Jiang A., Lin J., Li X. (2020). Data mining algorithms for bridge health monitoring: Kohonen clustering and LSTM pre-diction approaches. J. Supercomput..

[6] Mutlib N.K., Baharom S., El-Shafie A., Nuawi M.Z. (2015). Ultrasonic health monitoring in structural engineering: Buildings and bridges. Struct. Control Health Monit..

[7] Han Q., Xu J., Carpinteri A., Lacidogna G. (2014). Localization of acoustic emission sources in structural health monitoring of masonry bridge. Struct. Control Health Monit..

[8] Yamaguchi T., Hashimoto S. (2010). Fast crack detection method for large-size concrete surface images using percolation-based image processing. Mach. Vis. Appl..

[9] Tung P.-C., Hwang Y.-R., Wu M.-C. (2002). The development of a mobile manipulator imaging system for bridge crack inspection. Autom. Constr..

[10] Akagic A., Buza E., Omanovic S., Karabegovic A. Pavement crack detection using Otsu thresholding for image segmentation. Proceedings of the 2018 41st International Convention on Information and Communication Technology, Electronics and Microelectronics (MIPRO).

[11] Tang J., Gu Y. Automatic crack detection and segmentation using a hybrid algorithm for road distress analysis. Proceedings of the 2013 IEEE International Conference on Systems, Man, and Cybernetics.

[12] Ronny Salim L., La H.M., Zeyong S., Weihua S. Developing a crack inspection robot for bridge maintenance. Proceedings of the 2011 IEEE International Conference on Robotics and Automation.

[13] Kim J.-W., Kim S.-B., Park J.-C., Nam J.-W. Development of crack detection system with unmanned aerial vehicles and digital image processing. Proceedings of the 2015 World Congress on Advances in Structural Engineering and Mechanics (ASEM15).

[14] Chen Z., Li H., Bao Y., Li N., Jin Y. (2015). Identification of spatio-temporal distribution of vehicle loads on long-span bridges using computer vision technology. Struct. Control Health Monit..

[15] Ho H.-N., Kim K.-D., Park Y.-S., Lee J.-J. (2013). An efficient image-based damage detection for cable surface in cable-stayed bridges. NDT E Int..

[16] Chan B., Guan H., Jo J., Blumenstein M. (2015). Tuwards UAV-based bridge inspection systems: A reivew and an application perspective. Struct. Monit. Maint..

[17] Yang C.H., Wen M.C., Chen Y.C., Kang S.C. (2015). An optimized unmanned aeiral system for bridge inspection. Proceedings of the Insternational Symposium on Automation and Robotics in Construction.

[18] Dorafshan S., Maguire M., Hoffer N., Coopmans C. (2017). Fatigue Crack Detection Using Unmanned Aerial Systems in Un-der-Bridge Inspection.

[19] Yang Y.-S., Yang C.-M., Huang C.-W. (2015). Thin crack observation in a reinforced concrete bridge pier test using image processing and analysis. Adv. Eng. Softw..

[20] Song L., Wang X. (2021). Faster region convolutional neural network for automated pavement distress detection. Road Mater. Pavement Des..

[21] LeCun Y., Bottou L., Bengio Y., Haffner P. (1998). Gradient-based learning applied to document recognition. Proc. IEEE.

[22] Krizhevsky A., Sutskever I., Hinton G.E. Imagenet classification with deep convolutional neural networks. Proceedings of the Advances in Neural Information Processing Systems, Harrahs and Harveys.

[23] Simonyan K., Zisserman A. (2014). Very deep convolutional networks for large-scale image recognition. arXiv.

[24] Dorafshan S., Thomas R.J., Maguire M. (2018). Comparison of deep convolutional neural networks and edge detectors for im-age-based crack detection in concrete. Constr. Build. Mater..

[25] Zhang A., Wang K.C.P., Li B., Yang E., Dai X., Peng Y., Fei Y., Liu Y., Li J.Q., Chen C. (2017). Automated Pixel-Level Pavement Crack Detection on 3D Asphalt Surfaces Using a Deep-Learning Network. Comput. Civ. Infrastruct. Eng..

[26] Zhang A.A., Wang K.C.P., Fei Y., Liu Y., Tao S., Chen C., Li J.Q., Li B. (2018). Deep Learning–Based Fully Automated Pavement Crack Detection on 3D Asphalt Surfaces with an Improved CrackNet. J. Comput. Civ. Eng..

[27] Cha Y.J., Choi W., Büyüköztürk O. (2017). Deep learning-based crack damage detection using convolutional neural networks. Comput.-Aided Civ. Infrastruct. Eng..

[28] Xu Y., Bao Y., Chen J., Zuo W., Li H. (2018). Surface fatigue crack identification in steel box girder of bridges by a deep fusion convolutional neural network based on consumer-grade camera images. Struct. Health Monit..

[29] He K., Zhang X., Ren S., Sun J. Deep residual learning for image recognition. Proceedings of the IEEE Conference on Computer Vision and Pattern Recognition.

[30] Huang G., Liu Z., van der Maaten L., Weinberger K.Q. (2017). Densely Connected Convolutional Networks. Proceedings of the CVPR 2017, IEEE Conference on Computer Vision and Pattern Recognition.

[31] Long J., Shelhamer E., Darrell T. Fully convolutional networks for semantic segmentation. Proceedings of the IEEE Conference on Computer Vision and Pattern Recognition.

[32] Ronneberger O., Fischer P., Brox T. (2015). U-Net: Convolutional Networks for Biomedical Image Segmentation. Proceedings of the International Conference on Medical Image Computing and Computer-Assisted Intervention.

[33] Badrinarayanan V., Kendall A., Cipolla R. (2017). Segnet: A deep convolutional encoder-decoder architecture for image segmen-tation. IEEE Trans. Pattern Anal..

[34] Yang X., Li H., Yu Y., Luo X., Huang T., Yang X. (2018). Automatic Pixel-Level Crack Detection and Measurement Using Fully Convolutional Network. Comput. Civ. Infrastruct. Eng..

[35] Liu Z., Cao Y., Wang Y., Wang W. (2019). Computer vision-based concrete crack detection using U-net fully convolutional net-works. Autom. Constr..

[36] Ren Y., Huang J., Hong Z., Lu W., Yin J., Zou L., Shen X. (2020). Image-based concrete crack detection in tunnels using deep fully convolutional networks. Constr. Build. Mater..

[37] Li G., Ma B., He S., Ren X., Liu Q. (2020). Automatic Tunnel Crack Detection Based on U-Net and a Convolutional Neural Network with Alternately Updated Clique. Sensors.

[38] Chen F.-C., Jahanshahi M.R. (2018). NB-CNN: Deep Learning-Based Crack Detection Using Convolutional Neural Network and Naïve Bayes Data Fusion. IEEE Trans. Ind. Electron..

[39] Song W., Jia G., Zhu H., Jia D., Gao L. (2020). Automated Pavement Crack Damage Detection Using Deep Multiscale Convolutional Features. J. Adv. Transp..

[40] Wang X., Girshick R., Gupta A., He K. Non-local Neural Networks. Proceedings of the 2018 IEEE/CVF Conference on Computer Vision and Pattern Recognition.

[41] Vaswani A., Shazeer N., Parmar N., Uszkoreit J., Jones L., Gomez A.N., Kaiser Ł., Polosukhin I. (2017). Attention is all you need. Advances in Neural Information Processing Systems.

[42] Li X., Zhong Z., Wu J., Yang Y., Lin Z., Liu H. Expectation-maximization attention networks for semantic segmentation. Proceedings of the 2019 IEEE/CVF International Conference on Computer Vision (ICCV).

[43] Dung C.V., Anh L.D. (2019). Autonomous concrete crack detection using deep fully convolutional neural network. Autom. Constr..

[44] Bang S., Park S., Kim H., Kim H. (2019). Encoder–decoder network for pixel-level road crack detection in black-box images. Comput.-Aided Civ. Infrastruct. Eng..

[45] Mei Q., Gül M., Azim R. (2020). Densely connected deep neural network considering connectivity of pixels for automatic crack detection. Autom. Constr..

[46] Kingma D.P., Ba J. Adam: A method for stochastic optimization. Proceedings of the International Conference Learn. Represent (ICLR).

[47] He K., Zhang X., Ren S., Sun J. Delving deep into rectifiers: Surpassing human-level performance on imagenet classification. Proceedings of the IEEE International Conference on Computer Vision.

[48] Chen L.-C., Zhu Y., Papandreou G., Schroff F., Adam H. Encoder-decoder with atrous separable convolution for semantic image segmentation. Proceedings of the European conference on computer vision (ECCV).

[49] Choi W., Cha Y.-J. (2019). SDDNet: Real-Time Crack Segmentation. IEEE Trans. Ind. Electron..

